# Can differentiated care models solve the crisis in HIV treatment financing? Analysis of prospects for 38 countries in sub-Saharan Africa

**DOI:** 10.7448/IAS.20.5.21648

**Published:** 2017-07-21

**Authors:** Catherine Barker, Arin Dutta, Kate Klein

**Affiliations:** ^a^ Palladium, Americas Health Practice, Washington, DC, USA

**Keywords:** Differentiated care, ART, cost analysis, efficiency

## Abstract

**Introduction**: Rapid scale-up of antiretroviral therapy (ART) in the context of financial and health system constraints has resulted in calls to maximize efficiency in ART service delivery. Adopting differentiated care models (DCMs) for ART could potentially be more cost-efficient and improve outcomes. However, no study comprehensively projects the cost savings across countries. We model the potential reduction in facility-level costs and number of health workers needed when implementing two types of DCMs while attempting to reach 90-90-90 targets in 38 sub-Saharan African countries from 2016 to 2020.

**Methods**: We estimated the costs of three service delivery models: (1) undifferentiated care, (2) differentiated care by patient age and stability, and (3) differentiated care by patient age, stability, key vs. general population status, and urban vs. rural location. Frequency of facility visits, type and frequency of laboratory testing, and coverage of community ART support vary by patient subgroup. For each model, we estimated the total costs of antiretroviral drugs, laboratory commodities, and facility-level personnel and overhead. Certain groups under four-criteria differentiation require more intensive inputs. Community-based ART costs were included in the DCMs. We take into account underlying uncertainty in the projected numbers on ART and unit costs.

**Results**: Total five-year facility-based ART costs for undifferentiated care are estimated to be US$23.33 billion (95% confidence interval [CI]: $23.3–$23.5 billion). An estimated 17.5% (95% CI: 17.4%–17.7%) and 16.8% (95% CI: 16.7%–17.0%) could be saved from 2016 to 2020 from implementing the age and stability DCM and four-criteria DCM, respectively, with annual cost savings increasing over time. DCMs decrease the full-time equivalent (FTE) health workforce requirements for ART. An estimated 46.4% (95% CI: 46.1%–46.7%) fewer FTE health workers are needed in 2020 for the age and stability DCM compared with undifferentiated care.

**Conclusions**: Adopting DCMs can result in significant efficiency gains in terms of reduced costs and health workforce needs, even with the costs of scaling up community-based ART support under DCMs. Efficiency gains remained flat with increased differentiation. More evidence is needed on how to translate analyzed efficiency gains into implemented cost reductions at the facility level.

## Introduction

Global 90-90-90 targets and 2015 World Health Organization (WHO) guidelines call for universal access to and rapid scale-up in coverage of antiretroviral therapy (ART), at a time resources for HIV are constrained globally [1,2]. Donor HIV spending in low and middle-income countries declined by more than $1 billion in 2015 [3]. While domestic contributions have increased over the past decade, countries face barriers in terms of limited fiscal space, and human resources, infrastructure, and other health system constraints in scaling up HIV services [4–7]. With the need to treat more people and improve patient outcomes, developing countries and donors such as the U.S. President’s Emergency Plan for AIDS Relief (PEPFAR) are calling for efficiency gains that can achieve more with the resources available [8]. Some have suggested there is scope for improving efficiency due to wide differentials in observed unit costs of HIV interventions [9,10].

A path to greater ART efficiency may be adopting service delivery models that reduce use of health system inputs while maintaining or increasing quality of care and hence patient outcomes. In this context, differentiated care models (DCMs) for ART, explored in this paper, have been suggested to potentially maximize quality of care efficiently [11,12]. These models would adapt treatment guidelines to patient characteristics, such as the patient’s age, location, behaviour, and virological or immunological response to treatment. Under DCMs, ART patients who are stable require fewer clinical facility visits and laboratory tests, allowing health systems to focus resources on those more in need [13]. DCMs may combine multi-month scripting, where patients return to facilities at longer intervals, with community-based support for ART, such as adherence clubs or community antiretroviral (ARV) distribution points [13,14]. In South Africa, Zimbabwe, Swaziland, Zambia, and other countries, such DCMs are being piloted [15–17]. However, DCMs implemented at scale have not been evaluated or mathematically modelled. Potential short-term efficiency gains from scaling up DCMs may include savings in health worker time and facility use through reduced visits for stable patients, rationalized use of diagnostic testing for patient management, and reduced costs to the patient in terms of transportation and time waiting to see a provider. The latter may improve adherence by reducing the patient’s opportunity cost of acquiring ARV refills, especially if reinforcing messages are available in the community. Potential long-term cost savings stem from improved cohort-level outcomes such as reduced need for second-line therapy as patients adhere better to treatment and reduced need to conduct lost-to-follow-up tracing. These long-term benefits assume that quality of care is maintained or improved.

Research on DCMs so far has examined the feasibility of bringing programs to scale and the costs and patient outcomes in pilot-level implementation. A 2014 study in Malawi, Mozambique, South Africa and the Democratic Republic of the Congo showed the benefits of four approaches to simplifying ART delivery for stable patients; from the patient perspective, travel and lost income was reduced, and from the health system perspective, clinic attendance was improved and retention in care remained high [18]. There have been multiple studies of related community-level interventions to improve treatment outcomes [19–21]. For example, a recent cost-effectiveness study from South Africa concluded that adherence clubs were more cost-effective than conventional facility-based nurse-driven care [22].

While these studies suggest DCMs are cost-saving, no study projects the costs of increasing differentiation of ART service delivery across countries in an era of ART scale-up. This current study models the potential health system efficiency gains in terms of reduced facility-level costs and number of health workers needed for ART from implementing two types of DCMs compared to an undifferentiated care model in 38 sub-Saharan African countries from 2016 to 2020. We take into account underlying uncertainty in the projected numbers on ART and unit costs of interventions.

## Methods

### DCM conceptual framework

Currently, most low- and middle-income African countries have limited patient differentiation in ART service delivery. Frequency of facility visits or laboratory testing is not necessarily based on response to treatment or other patient characteristics, and most ART services are offered in facility-based settings [18]. As countries expand access to treatment to all people living with HIV (PLHIV), such undifferentiated care may not be sustainable nor yield the best outcomes [13]. As discussed, DCMs may allow more people to be on treatment with the resources available, and respond to a need for patient-centred care [13,18].

The 2016 WHO ART guidelines recommend differentiating four groups: PLHIV presenting for care at earlier stages of the disease, PLHIV presenting with advanced disease, patients stable on ART, and those unstable [11]. The package of care for stable patients on ART – defined by WHO as those receiving ART for at least one year with no adverse drug reactions, no current opportunistic infections or pregnancy, a good understanding of lifelong adherence, and evidence of treatment success – includes less frequent clinical and refill visits. Unstable patients may need specialized care, enhanced adherence support, additional viral load testing, changes in ART regimen, or monitoring for HIV drug resistance [11]. In line with previous guidelines, WHO makes specific recommendations for children and adolescents, pregnant women, and people with co-morbidities, including tuberculosis. Treatment among these groups may differ from the standard recommendations for each of the four differentiated groups. For instance, children and adolescents who are experiencing rapid growth, even if responding well to treatment, need more frequent monitoring than adults for treatment dosing changes and adherence support [11].

In addition to the WHO guidelines, donors and global organizations, including PEPFAR; the Global Fund to Fight AIDS, Tuberculosis, and Malaria; UNAIDS; and Medécins Sans Frontières, have issued guidance on differentiated care [13,14,23,24]. Some countries, including Zimbabwe, Kenya, Swaziland, and South Africa, have developed country-specific guidelines adopting aspects of differentiated care [16,25–27]. A conceptual framework comparing differentiated care and current practice in terms of the ranges in frequency of clinical and medication visits, frequency and type of laboratory testing, and level of community-based ART is shown in Table 1.

**Table 1.** Differentiated care conceptual framework: frequency and types of visits, lab tests, and community-based ART

		Patient-centred differentiated care based on …							
		Demographics				Health status and clinical characteristics			
	Current model, limited differentiated care	Age	Sex	Key populations	Urban vs. rural	Pregnant and postpartum women	Treatment stability	Comorbidities	Regimen type
**Clinical visits**	4-12 visits/year, varies by country standards and guidelines	Children 0-9: 4-12 visits/year; monthly visits up to 18 months due to rapid growth; should coincide with clinical visits of other family members Adolescents 10-19: 2-6 visits/year; those on adult doses can be seen less frequently than those on paediatric doses; may have specific clinic days or school-based program Adults 20+: 1-6 visits/year	Services offered alongside ART may vary by sex; for example, males and females may receive different types of sexual and reproductive health (SRH) services and NCD services	At least 4 clinical visits/year; integrated with SRH and population-specific services	No difference based on regimen	4-12 visits/year, linked to ANC and PNC; may vary based on timing of diagnosis; requires additional counselling	New: 4-6 visits/year Stable: 1-4 visits/year Unstable: 6-12 visits/year	Clinical visits should be integrated with other services (e.g., TB), visits may be more frequent and require counselling	No difference based on regimen
**Refill visits**	4-12 visits/year, usually linked to clinical visits	Children and adolescents: 4-12 visits/year, linked to clinical visits Adults: 2-6 visits/year, depends on stability, location, and other factors; may be linked to clinical visits		At least 4 refill visits/year	Urban patients may be closer to a health facility and can have more frequent refill visits than rural patients	4-12 visits/year, linked to clinical visits	2-4 visits/year for new or stable patients; de-linked to clinical visits and “fast-tracked”	Should be able to collect all drugs needed at the same facility on the same day to avoid additional visits	New regimens, particularly self-injecting ARVs, may change number of refill visits in the future
**Laboratory testing**	Annual viral load (where available), haematology, and clinical chemistry panel tests; CD4 tests twice/year	Viral load twice/year for children regardless of stability and unstable adolescents and adults, once/year for stable adolescents and adults; No CD4 tests, limited haematology or clinical chemistry panel tests (based on 2016 WHO recommendations)		Annual viral load; May receive additional STI testing compared with other adults; No CD4 tests, limited haematology or clinical chemistry panel tests	No difference in testing	2-6 viral load tests/year, depending on viral suppression status; No CD4 tests, limited haematology or clinical chemistry panel tests	Viral load twice/year for new and unstable; once/year for stable; No CD4 tests, limited haematology or clinical chemistry panel tests	May receive additional testing related to co-morbidities	Annual creatinine test (TDF-containing regimens), annual haemoglobin test (AZT-containing regimens); patients switching to second line treatment may have additional viral load tests
		Community-based support for ART that scales up under differentiated care							
**Community-based support for ART**	Small-scale programs available in certain communities; can involve facility staff, community health workers, and peer educators; includes ART adherence clubs, ARV distribution points, and other types of community support; membership size and frequency of meetings depend on type of support and vary by country	Children: Community education and support for caretakers Adolescents: Teen clubs, peer support Adults: Stable patients can join adherence clubs and ARV pick-up/distribution groups in place of clinical visits	Community-based peer support groups may be female or male only, especially if linked to PMTCT	Peer support and adherence community-based programs; can be linked to prevention outreach and behavioural interventions	Type of community group may vary; for instance, ARV distribution may be more appropriate for rural rather than urban areas	Peer support groups specifically for pregnant and breastfeeding women living with HIV	Only stable adult patients are eligible to receive community-based ART in place of clinical visits, but new and unstable patients may also be involved in community-based education and adherence support	Patients with comorbidities are not eligible to participate in community-based ART in place of clinical visits	No difference based on regimen

Based on this conceptual framework, we developed three stylized service delivery models for analysis (Table 2). The first reflects an undifferentiated care model, assuming that all groups receive the same number of visits and types of lab tests based on average current practice. This model excludes the costs of community-based ART as this is not currently offered at scale. The second model is a DCM in which care varies by age and stability and community-based ART is scaled up. The final model is a four-criteria DCM, which includes the same differentiation as the age and stability DCM as well as differentiation for key populations and urban versus rural populations. While these models may not be fully representative of service delivery in the 38 countries, they illustrate potential options for countries’ consideration. In order to maximize efficiency under differentiated care, viral load monitoring should coincide with clinic visits.

**Table 2.** Service delivery models and assumptions by ART patient group

			Model 3: Four-criteria DCM (differentiation by age, stability, key population, and urban/rural)											
			Model 2: Age and stability DCM											
			Children 0–9			Adolescents 10–19			Adults 20±					
Frequency per year or percentage receiving community-based support for ART		Model 1: Undifferentiated care	*New*	*Stable*	*Unstable*	*New*	*Stable*	*Unstable*	*New*	*Stable*	*Unstable*	Key populations^a^	Urban^b^	Rural^b^
**Clinical visits**		6	6	4	6	5	3	6	4	2	6	4-6	2-6	2-6
**Refill visits**		6	4	3	6	4	3	6	4	2	6	4-6	3-7	1-5
Lab testing*	*Viral load*	1	2	2	2	2	1	2	2	1	2	1	1-2	1-2
	*CD4*	2	0	0	0	0	0	0	0	0	0	0	0	0
	*Clinical chemistry*	1	1*	1*	1*	1*	1*	1*	1*	1*	1*	1*	1*	1*
	*Haematology*	1	1**	1**	1**	1**	1**	1**	1**	1**	1**	1**	1**	1**
**Coverage of community-based ART support or home visits**		0%	100%	100%	100%	100%	100%	100%	0	100%	0	100%	Lower cost	Higher cost

*Clinical chemistry tests are for those on TDF-containing regimens; **haematology tests are for those on AZT-containing regimens. ^a^Key populations for this analysis are defined as men who have sex with men, sex workers, and people who inject drugs. For our analysis, key populations are a subset of the adult population only. New and stable key populations have 4 visits per year, unstable key populations have 6. ^b^The entire population can be segmented in urban vs. rural. Due to closer proximity to facilities in urban areas, the model assumes additional refill visits and fewer community ART support meetings for those residing in urban areas compared with rural areas.

### Cost and full-time equivalent health worker analyses

For each model, we estimated the total costs of ARV drugs, laboratory commodities, facility-level personnel and overhead, and community-based ART support across 38 sub-Saharan Africa countries from 2016 to 2020 (Table 3). We compare the undifferentiated care model to the two DCMs to estimate the potential cost and health worker savings of implementing DCMs. We estimated the number of full-time equivalent facility-level health workers needed for each model based on previously collected estimates of how much time different cadres spend delivering ART services in Africa, accounting for current task sharing practices [28,29].

**Table 3.** Countries included by income level and region

	Eastern and Southern Africa (AES)	West and Central Africa (WCA)
**Low income (LIC)**	Burundi, Eritrea, Madagascar, Malawi, Mozambique, Rwanda, South Sudan, Tanzania, Uganda, Zimbabwe	Benin, Burkina Faso, Central African Republic, Chad, Democratic Republic of the Congo, Gambia, Guinea-Bissau, Liberia, Mali, Togo
**Lower-middle income (LMIC)**	Kenya, Lesotho, Swaziland, Zambia	Cameroon, Congo, Cote d’Ivoire
**Upper-middle or high income (UMIC/HIC)**	Angola, Botswana, Mauritius, Namibia, South Africa	Equatorial Guinea, Gabon, Ghana, Mauritania, Nigeria, Senegal

We modelled underlying uncertainty in the numbers of people on ART and the unit cost of treatment inputs per person. We conducted probabilistic sensitivity analysis and expected mean values were derived from sampling events in 5000 second-order Monte Carlo simulation trials performed using RiskAMP software (Structured Data LLC) integrated with Microsoft Excel [30]. Each simulation was run simultaneously over all uncertain parameters. The simulation utilized distribution types with bounds and modes derived from country- or regional-specific secondary data as described in the Supplementary File.

#### Estimating numbers on treatment by sub-population

The study estimated the number of children (ages 0-9), adolescents (ages 10–19), and adults (ages 20 and above) living with HIV from 2016 to 2020 in each country using projections from the uncertainty analysis tool within the Spectrum AIDS Impact Model (AIM) (see Supplementary File 1) [6]. The AIM analysis assumed all PLHIV are eligible for treatment. The number of PLHIV was multiplied by country-specific coverage rates (e.g., the percentage of all PLHIV on treatment) to estimate numbers on treatment. Baseline coverage estimates from December 2015 for children under 15 and adults 15 and older were from the UNAIDS AIDSinfo database [31]. Annual increases in coverage were based on reaching the second 90 target of 81% of all PLHIV on treatment by 2020 [1]. Countries with an adult ART coverage rate below 40% in 2015 were assumed to reach the second 90 in 2025 rather than 2020.

For the DCMs, the age-disaggregated number of people on ART were further divided into three categories – new, stable established, and unstable established patients – based on country- or regional-specific data on retention, mortality, and viral load suppression [31–33]. We assumed retention rates will improve based on studies showing improvement in retention after introducing DCMs, and that mortality rates will decline from 2016 to 2020 in line with mortality rate declines among PLHIV in sub-Saharan Africa from 2010 to 2015 [18,34]. In the four-criteria DCM, the number of adults on ART were further disaggregated into general or key population based on country- or regional-specific data on population size, HIV prevalence, and ART coverage estimates for men who have sex with men, sex workers, and people who inject drugs [31,32,35–39]. The analysis assumes ART coverage among key populations increases over time. The four-criteria DCM also disaggregates all patients into urban versus rural based on the country-specific percentages of people residing in urban areas, weighted by the difference in HIV prevalence in urban versus rural areas [32,40,41]. See Supplementary File 1 for assumptions on the parameters for population disaggregation.

#### Estimating ART unit costs

We accounted for uncertainty in unit costs for ARVs, laboratory tests, facility-based personnel and overhead using probabilistic sensitivity analysis based on ranges for current baseline costs and future reductions in costs (ARVs and laboratory only). Cost data were adjusted to constant 2015 US dollars [42]. ARV regimen costs, costs per laboratory test, and personnel and overhead costs per visit are the same across all scenarios in the analysis, meaning any difference in total costs over time is attributable to differences in the service delivery models rather than cost inputs into the model.

The uncertainty analysis parameters for ARV costs were based on 2015 adult, adolescent and pediatric first- and second-line ARV costs, separated by income level and region, from the Global Price Reporting Mechanism (GPRM) database [43]. The GPRM yields the lowest, median, and highest cost per patient-year by regimen and dosage strength based on country-specific transactions. The regimens chosen for analysis and the proportions of patients on each regimen annually were based on WHO recommendations and ARV market analyses [43,44].

We modelled the unit cost of laboratory tests as uncertain based on recent literature on the reagent and consumables costs of viral load, CD4 cell count, haematology and clinical chemistry panel tests. Costs per test from 12 countries, categorized by country income level, were used in the analysis. Overall laboratory costs per patient per year (Table 4) were based on frequency assumptions which varied by patient group, as shown in Table 2 [29,45–50].

**Table 4.** Mean ARV and laboratory testing costs per person-year

	LIC		LMIC		UMIC/HIC	
**ARVs**	**2016**	**2020**	**2016**	**2020**	**2016**	**2020**
Adults and adolescents (SEA)	$110	$99	$102	$97	$136	$126
	($91-133)	($80-120)	($90-117)	($82-112)	($88-203)	($85-184)
Adults and adolescents (WCA)	$109	$98	$109	$105	$124	$113
	($85-132)	($77-120)	($98-122)	($90-120)	($115-134)	($98-125)
Children (SEA)	$134	$102	$122	$93	$299	$292
	($123-146)	($84-120)	($114-131)	($76-113)	($283-314)	($240-333)
Children (WCA)	$158	$119	$171	$128	$168	$157
	($133-187)	($91-149)	($162-180)	($107-150)	($160-177)	($138-174)
**Lab**						
Undifferentiated care	$42	$34	$41	$33	$48	$39
	($30-56)	($23-47)	($29-54)	($22-46)	($32-67)	($25-56)
Differentiated: children	$52	$39	$45	$33	$53	$40
	($27-78)	($18-65)	($28-60)	($18-51)	($26-90)	($18-73)
Differentiated: stable	$27	$21	$24	$18	$28	$22
	($15-41)	($11-34)	($15-32)	($10-27)	($15-47)	($11-$39)
Differentiated: unstable	$53	$40	$46	$34	$54	$41
	($27-$79)	($19-66)	($28-61)	($19-52)	($27-91)	($19-75)

95% confidence intervals are in parentheses. Confidence interval and mean generated from probabilistic sensitivity analysis.

Based on a review of recent country-specific studies, we estimated ranges for the cost per clinical and ARV refill visit of facility-level overhead (e.g., for utilities) and personnel costs (e.g., for health workers who directly deliver treatment interventions). Data from 10 studies across 11 countries were included in the analysis [50–58]. We assumed the unit costs would be higher as country income levels increased. Overhead and personnel costs per patient per year were estimated by patient group based on the frequency of clinical and ARV refill visits per year.

Few studies estimate community-based ART costs. Community-based ART cost ranges were derived from four studies [15,55,59,60]. The analysis assumes community-based ART programs cost more in rural than urban areas [13]. The unit cost ranges used for probabilistic sensitivity analysis are wide, as costs depend on the type of support offered (e.g., ARV distribution point vs. adherence support group) and personnel involved (e.g., facility-based health workers vs. peer volunteers) [59].

## Results

**Figure 1.**
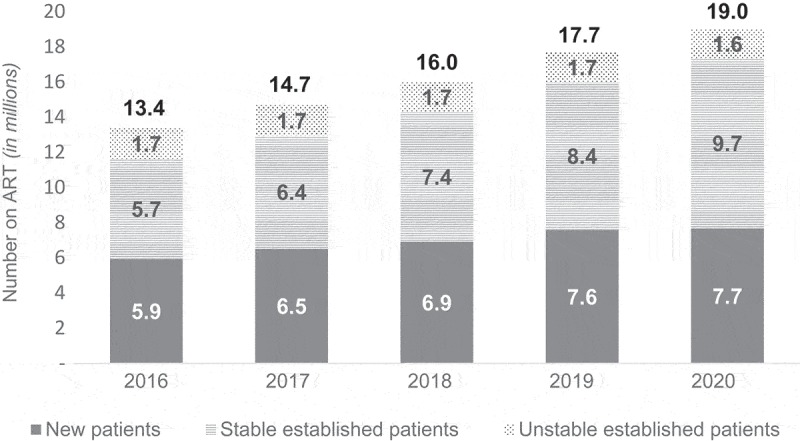
Projections of number of people on ART annually. The number of people on ART is assumed to be the same across all three models. This graph shows the mean annual estimates disaggregated by stability on ART. The proportion of established ART patients who are stable is projected to increase from 43% to 51% from 2016 to 2020. The percentage of patients who are new is estimated to decline from 45% in 2016 to 40% in 2020. Similarly, the percentage of established patients who are unstable declines from 13% to 9% over the same time period.

We estimate the number of children ages 0 to 9 on ART will decrease from 0.48 million (95% confidence interval [CI]: 0.46-0.51 million) in 2016 to 0.42 million (95% CI: 0.40-0.44 million) in 2020 due to increased prevention of mother-to-child transmission, resulting in decreases in the future population of children living with HIV. The number of adolescents and adults on ART is estimated to increase from 12.9 million (95% CI: 12.6-13.2 million) in 2016 to 18.6 million (95% CI: 18.0-19.1 million) in 2020. The percentage of people on ART who are stable, established patients are estimated to increase over time (Figure 1). Key populations represent 2% of adults on ART each year. The analysis estimates that 51% of people on ART in 2020 will reside in urban areas, a slight increase from 48% in 2016.

**Figure 2.**
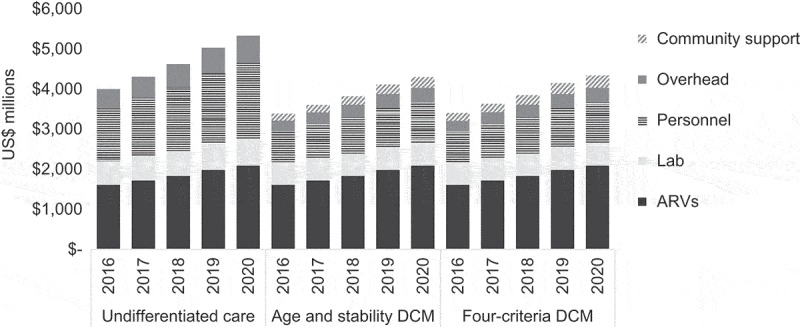
Annual ART costs by model and cost category. This chart shows the annual mean costs of each model. Facility-based costs increase by 34% from 2016 to 2020 under the undifferentiated care model. Costs are lower under the DCMs. Under the DCMs, facility- and community-based costs are estimated to increase by 28% from 2016 to 2020.

Under the undifferentiated care model, total five-year facility-based ART costs are estimated to be US$23,305 million (95% CI: $23,273-$23,505 million). An estimated $5123 million (95% CI $5109-$5203 million) and $5,122 million (95% CI: $5108-$5201 million) in facility-based ART costs could be saved from 2016 to 2020 from implementing the age and stability DCM and four-criteria DCM, respectively. Differentiating care to certain groups requiring more intensive inputs under the four-criteria DCM prevents further efficiency gain compared to the age and stability DCM. When adding community-based ART support needed under DCMs, total cost savings from implementing DCMs decline slightly, but are still large with an estimated cost reduction of 17.5% (95% CI: 17.4-17.7%) and 16.8% (95% CI: 16.7-17.0%) under the age and stability DCM and four-criteria DCM, respectively. Annual cost savings from DCMs are estimated to increase over time (Figure 2).

Both DCMs result in similar levels of facility-level cost savings. Although the four-criteria model calls for increased clinical and refill visits for key versus general population adults, key populations represent a small proportion (2%) of all adults on ART and overall costs. Also, the cost savings from assumed lower refill visits for rural ART patients are nearly equal to the additional costs from assuming that urban ART patients will maintain more frequent refill visits. When factoring in community support costs, the difference in cost savings between the two DCMs becomes significant. This is a result of assuming a lower unit cost for community ART support among urban patients, who represent the majority of those on ART, compared with rural patients. We assumed urban ART patients who likely have better access to health facilities than those in rural areas would require fewer community ART support meetings and ARV distribution points.

Cost savings in facility-based service delivery from DCMs are driven by a 44% reduction in overhead and personnel costs and an 11% reduction in laboratory costs. The laboratory cost savings assume CD4 count testing is discontinued under DCMs; however, even if annual CD4 testing was continued for 10-35% of those on ART with poor immune reconstitution, DCMs would save $176-$298 million in laboratory costs across all five years [61]. ARV costs are the same across all models, but account for 40% of total five-year costs in the undifferentiated care model vs. 48% of total costs in the DCMs. Adult patients represent the bulk (91%) of facility-based costs in the age and stability DCM from 2016 to 2020. New, stable, and unstable patients account for 48%, 38%, and 14% of the total five-year costs, respectively, in this model. Costs are split evenly across urban (51%) and rural (49%) populations in the four-criteria DCM. Just 2% of total facility-based costs are for key populations.

**Figure 3.**
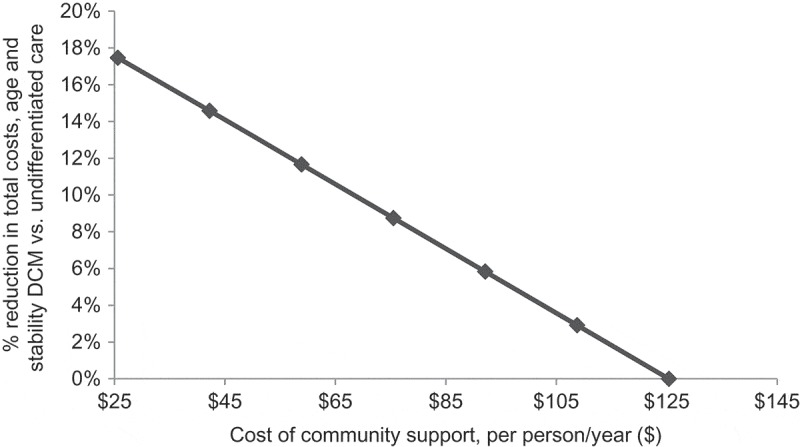
Community-based ART support: cost acceptability curve. This chart shows the percentage cost savings from implementing the age and stability DCM compared to undifferentiated care (*y*-axis) as community-based ART unit costs increase (*x*-axis).

Due to the potentially wide range in community-based ART unit cost, we estimated the relationship between this unit cost and the savings from implementing DCMs, using the age and stability DCM as an example (Figure 3). Community-based ART support interventions could cost as much as $125 per person per year before they negate the cost savings from implementing differentiated care.

**Figure 4.**
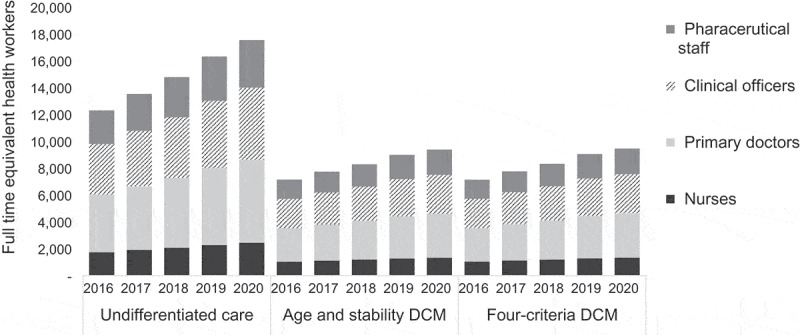
Number of full-time equivalent health workers needed for ART by model, year, and type of health worker. This chart shows the mean estimated number of health workers needed to deliver ART services each year by service delivery model and type of health worker. Under the undifferentiated care model, the number of health workers needed for ART is estimated to increase by 43%, compared to 31% and 32% in the age and stability DCM and four-criteria DCM, respectively.

DCMs also decrease the full-time equivalent (FTE) health workforce requirements for ART (Figure 4). An estimated 8159 (95% CI: 4975-12,191), or 46.4% (95% CI: 46.1-46.7%), fewer FTE health workers across critical cadres are needed in 2020 for the age and stability DCM compared with undifferentiated care. This reduction is driven by a decrease in need for primary care doctors (36% of the reduction), clinical officers (30%), pharmaceutical staff (20%), and nurses (14%).

## Discussion

A previous analysis suggested that Eastern and Southern Africa alone would account for 61% of the global financing gap for facility-based ART over 2016-2020 [6]. Using that study’s data, we estimate that the median financing gap as a proportion of total costs after considering domestic and external contributions specifically for the 38 countries in this analysis was 46% for Eastern and Southern Africa and 52% in West and Central Africa, while the minimum gap ranged from 12-16% [6]. Cost categories considered were the same across studies. Our findings suggest that the implementation of DCMs in sub-Saharan Africa could yield total reductions in cost of health system inputs of 17-18%. Using the median gap as a benchmark, this means that DCMs could reduce the financing gap for facility-based ART services by 32-36%, even after accounting for additional community-based ART support costs, due to reduction in personnel, overhead, and laboratory costs. Previously, it has been estimated that ARV price reductions and efficiencies in dosing could eliminate half of the financing gap over 2016-2020 if successfully implemented [6]. Therefore, ARV cost reductions combined with DCMs hold substantial promise for improving the sustainability of scaled-up HIV treatment in Africa.

A practical implication of such efficiency gains is that additional ART patients could be managed with existing health resources and future needs would be reduced for investments in infrastructure or new health workers related to HIV treatment. Given competing priorities for government resources in the Sustainable Development Goals era, this is very desirable. Governments can then distribute increases in health spending across needs in multiple health domains. However, for our analysed gains in efficiency to translate into real reductions in additional health system resources needed as patient numbers increase, some conditions must be met. First, health worker time management at the facility level is critical. Initially, as the volume of patients’ clinic visits reduces once DCMs are implemented – that is, before more patients can be associated with each facility given ongoing scale-up towards 90-90-90 targets – “slack” in health worker capacity must be taken up. If certain health workers are pre-assigned and cannot be shifted to other interventions, for example, at standalone ART clinics, even those now not crowded, then efficiency gains are not readily realizable. Second, overall reduced use of facility overhead notionally allocated to ART can appear intangible to facility managers. Practically, only reduced crowding and waiting time at outpatient departments and reduced cost of consumables related to lower patient flow can be realized into tangible changes. For example, facility managers can increase or adjust clinic hours to allow more patients of other health areas to be seen.

Gains in the efficiency of use for health system inputs such as health worker time and facility space and overhead accrue to the facility operators involved in ART services. There must be political will and policy flexibility to make meaningful adjustments in response to realizable efficiency gains.

Implementing new service delivery models often require changes to guidelines, with stakeholder consultation and initial piloting. Health workers and service delivery managers require retraining in the revised processes and on identifying different patient groups appropriately for differentiation. Community-based interventions may require additional upfront and recurring investments to ensure that they yield the best outcomes for adherence and patient management. Identifying and keeping updated registers of stable vs. unstable patients, general vs. key population PLHIV, and other patient disaggregation require investments in information systems, including laboratory systems and epi-behavioural surveillance. Additional research is needed to understand DCM start-up costs as they are not estimated in this study; however, some of these costs could be absorbed by ongoing in-service training programs and periodic revision of service delivery guidelines. A limitation of our analysis is the focus on the health system funders’ perspective. We did not estimate costs from a full economic lens, which would include items such as transportation and opportunity costs faced by patients and caregivers under DCMs vs. undifferentiated care. The reductions in such costs may be significant. The overarching rationale for our analysis is that in addition to efficiency gains, DCMs may yield long-term benefits by improving patient management and hence health outcomes; however, this was not based on any modelling analysis.

## Conclusions

Our results suggest that there may be considerable basis for the widespread adoption of DCMs from a cost-efficiency and health workforce optimization standpoint. This argument is further strengthened if DCMs are beneficial to long-term health outcomes for patients by identifying those requiring specialized and/or more intensive care approaches. More evidence is needed on cost and cost-efficiency aspects of community-based ART support interventions that should be paired with DCMs. Our study is the first systematic and multi-country examination of the potential efficiency considerations of such DCMs – more analyses are needed using real-world data and to include other high HIV burden regions.

## Notes

To access the supplementary material to this article please see Supplementary Files under Article Tools online.
